# Bioglass and Vitamin D3 Coatings for Titanium Implants: Osseointegration and Corrosion Protection

**DOI:** 10.3390/biomedicines11102772

**Published:** 2023-10-12

**Authors:** Irina Negut, Gratiela Gradisteanu-Pircalabioru, Mihaela Dinu, Bogdan Bita, Anca Constantina Parau, Valentina Grumezescu, Carmen Ristoscu, Mariana Carmen Chifiriuc

**Affiliations:** 1 National Institute for Laser, Plasma and Radiation Physics, 409 Atomistilor Street, P.O. Box MG 36, 077125 Magurele, Romania; negut.irina@inflpr.ro (I.N.); bogdan.bita@inflpr.ro (B.B.); valentina.grumezescu@inflpr.ro (V.G.); 2 eBio-Hub Research Center, University Politehnica of Bucharest—CAMPUS, 6 Iuliu Maniu Boulevard, 061344 Bucharest, Romania; ggradisteanu@upb.ro; 3 Research Institute of the University of Bucharest (ICUB), University of Bucharest, 050657 Bucharest, Romania; carmen.chifiriuc@bio.unibuc.ro; 4 Academy of Romanian Scientists, 3 Ilfov Str., District 5, 050044 Bucharest, Romania; 5 National Institute of Research and Development for Optoelectronics-INOE2000, 409 Atomistilor St., 077125 Magurele, Romania; mihaela.dinu@inoe.ro (M.D.); anca.parau@inoe.ro (A.C.P.); 6 Faculty of Physics, University of Bucharest, 077125 Magurele, Romania; 7 The Romanian Academy, Calea Victoriei 25, District 1, 010071 Bucharest, Romania; 8 Department of Microbiology, Faculty of Biology, University of Bucharest, 050095 Bucharest, Romania

**Keywords:** bioglass, cytotoxicity, laser deposition, MAPLE, thin films, vitamin D3

## Abstract

The use of MAPLE synthesized thin films based on BG and VD3 for improving the osseointegration and corrosion protection of Ti-like implant surfaces is reported. The distribution of chemical elements and functional groups was shown by FTIR spectrometry; the stoichiometry and chemical functional integrity of thin films after MAPLE deposition was preserved, optimal results being revealed especially for the BG+VD3_025 samples. The morphology and topography were examined by SEM and AFM, and revealed surfaces with many irregularities, favoring a good adhesion of cells. The thin films’ cytotoxicity and biocompatibility were evaluated in vitro at the morphological, biochemical, and molecular level. Following incubation with HDF cells, BG57+VD3_ 025 thin films showed the best degree of biocompatibility, as illustrated by the viability assay values. According to the LDH investigation, all tested samples had higher values compared to the unstimulated cells. The evaluation of cell morphology was performed by fluorescence microscopy following cultivation of HDF cells on the obtained thin films. The cultivation of HDF’s on the thin films did not induce major cellular changes. Cells cultured on the BG57+VD3_025 sample had similar morphology to that of unstimulated control cells. The inflammatory profile of human cells cultured on thin films obtained by MAPLE was analyzed by the ELISA technique. It was observed that the thin films did not change the pro- and anti-inflammatory profile of the HDF cells, the IL-6 and IL-10 levels being similar to those of the control sample. The wettability of the MAPLE thin films was investigated by the sessile drop method. A contact angle of 54.65° was measured for the sample coated with BG57+VD3_025. Electrochemical impedance spectroscopy gave a valuable insight into the electrochemical reactions occurring on the surface.

## 1. Introduction

The most popular implant materials are titanium (Ti) and its alloys, due to their outstanding combination of properties, such as strong corrosion resistance, advantageous mechanical qualities, and biocompatibility [1]. Despite the high success rates of Ti implants, some issues can arise, such as immediate (contact dermatitis and eczema) or delayed hypersensitivity, as well as other immunological reactions [2]. Moreover, it is well-known that one of the causes of implant failure is the existence of previous conditions such as osteoporosis or other bone-related issues [3,4]. While Ti is generally corrosion-resistant, it can be susceptible to localized corrosion in specific environments, such as those with high chloride ion concentrations [5]. This can lead to the formation of pits or crevices on the implant’s surface, potentially compromising its structural integrity [6]. Ti implants are often passivated to enhance their corrosion resistance. Passivation involves the formation of a stable oxide layer on the implant’s surface. If the passivation process is inadequate or compromised, the implant may become more susceptible to corrosion [7]. In some cases, biological factors can contribute to corrosion-related Ti implant failure. These may include the presence of specific enzymes, proteins, or ions in the body that can affect the corrosion resistance of Ti.

Numerous skeletal outcomes, including osteoporotic fractures [8], impaired fracture healing [9] and paradontosis [10] are linked to vitamin D insufficiency. Extreme vitamin D deficiency significantly raises the risk of infections and, in extreme cases, even death [11]. In the human body, the modulation of cell development, neuromuscular transmission, and inflammatory processes are a few physiological processes influenced by the presence of vitamin D. Dysbasia, i.e., impaired movement and balance, and a higher incidence of falls, particularly those that result in fractures, are the most often documented consequences of vitamin D insufficiency [12,13]. In response, surgeons and healthcare guidelines advise the prescription of vitamin D3 (VD3) supplements to patients, including those who are healthy and non-osteoporotic, to improve the results of fracture healing, prevent osteoporosis and maintain overall bone health [14].

On the materials science side, to ensure the long-term integration of implants into the body, it is necessary to continuously improve their properties by the design of implant materials. One method for increasing the implants’ corrosion resistance, biocompatibility, and bioactivity is surface functionalization, which has recently attracted a lot of attention [15,16,17]. Chemical modification, ion implantation, anodic oxidation, physical vapor deposition, chemical vapor deposition, plasma spray deposition, sol-gel, thermal oxidation, laser deposition are just some examples of techniques which have been used for the application of composite coatings to modify Ti implant-like surfaces [16,17]. An inexpensive and effective procedure to obtain coatings in order to improve the bioactivity and corrosion resistance of Ti implant-like surfaces is the Matrix Assisted Pulsed Laser Evaporation (MAPLE) technique [18]. 

It has been previously shown that the presence of inorganic coatings on implants, such as bioglass (BG) [19,20,21], calcium phosphates [22], and hydroxyapatite [23], stimulates and speeds up bone development. BGs are specialized materials with a unique and tunable composition that promotes bone regeneration, possesses excellent biocompatibility, exhibits bioactivity, and is extensively used in medical and dental applications [24]. The solubility of BG in the human environment is a critical property that contributes to its effectiveness in bone repair [25]. BGs are designed to be bioactive, which means that they have the ability to dissolve and interact with the biological environment when implanted in the body [26]. By gradually dissolving and releasing the bioactive ions from its composition (e.g., Si, Ca, Na, P), BG creates an environment conducive to bone regeneration, making it a valuable material in orthopedic implants, bone grafts, and tissue engineering for bone repair [25,26]. The released ions can stimulate the formation of a mineralized tissue layer on the surface of the BG. This layer primarily consists of hydroxyapatite, which is a natural component of bone [27]. The gradual dissolution of BG allows for a strong integration between the implant and the surrounding bone tissue [25,26]. This integration is vital for the long-term success of orthopedic implants or bone graft materials.

Due to the bone-bonding ability of BG, BG coatings have been used to treat/reconstruct bones affected by various bone diseases and to strengthen the linkages between the bone and implant [18]. In our prior research, we established that the MAPLE deposition method is well-suited for fabricating thin films with BG that promote biocompatibility and bone healing. These films were skillfully engineered to exhibit exceptional biocompatibility with different cell lines [28,29,30].

Taking into account the above mentioned, this paper endeavors to investigate the applicability of the MAPLE deposition technique to obtain functionalized Ti implant-like surfaces for medical applications where the osseointegration process is paramount. In this regard, our primary objectives in this study encompass: (i) improving the osteointegration of Ti-implant-like surfaces, (ii) increasing the corrosion resistance of Ti-implant-like surfaces when exposed to aggressive conditions and (iii) appraising the cytotoxicity of the thin films concerning host cells. In this study, we synthesized a composite thin film consisting of BG and VD3 using the MAPLE technique onto Ti implant-like surfaces.

### Statement of Significance

The novelty of this research lies in obtaining thin films by MAPLE from two different materials: BG, renowned for its bioactive properties and the ability to stimulate bone growth, and VD3, a vital nutrient known for its role in bone health. In this study, we delve into the properties and performance of these thin films, examining their in vitro responses, particularly in the context of Ti implant osseointegration. 

The primary objective was to improve the osteointegration of Ti-implant-like surfaces by means of BG+VD3 thin films while concurrently increasing their corrosion resistance when exposed to aggressive conditions (human body fluids). 

Thin films offer distinct advantages, such as precise control over material deposition and the potential for targeted delivery of therapeutic agents. Through the innovative use of MAPLE, we have been able to create these thin films, opening new avenues for exploration in the field. 

In summary, this study breaks new ground by conducting a thorough assessment of thin films derived from BG and VD3, shedding light on their potential applications in bone tissue engineering. 

## 2. Materials and Methods

### 2.1. Materials

Chloroform (CHCl_3_), analytical grade acetone (C_6_H_6_O), ethanol (C_2_H_5_OH), and reagents required for the preparation of the simulated body fluid (SBF), such as NaCl, NaHCO_3_, KCl, K_2_HPO_4_·3H_2_O, MgCl_2_·6H_2_O, HCl, CaCl_2_, Na_2_SO_4_, and (CH_2_OH)_3_CNH_2_ were acquired from Sigma-Aldrich Chemie GmbH (Steinheim, Germany). The SBF with an ionic composition similar to that of blood plasma was prepared taking into account the corrected Kokubo SBF recipe [28] by mixing the substances and following the same order and quantities. Dimethyl sulfoxide (DMSO) was purchased from Sigma-Aldrich. 

Grade 4 titanium (Ti) foil of (0.8 × 0.8) cm^2^ and Si (100) plates were used as deposition substrates. The preparation of the deposition substrates followed a protocol: cleaning was performed for 15 min in acetone, ethyl alcohol, and deionized water, respectively, in an ultrasonic bath (Elma Schmidbauer GmbH, Singen, Germany), before drying in a stream of N_2_. Afterwards, the cleaned substrates were fixed onto a support, to be inserted into the reaction chamber. Ti was chosen because of its well-established compatibility with bone, which explains its extensive use as a biomaterial for implantable devices [31]. For dedicated cross-section SEM investigations, Si wafers of (1 × 1) cm^2^ were used. The same cleaning and deposition protocols were applied. 

BG powders with a SiO_2_ concentration of ~57% (further denoted as BG57) and a compositional system of SiO_2_–Na_2_O–K_2_O–CaO–MgO–P_2_O_5_ to obtain the targets was employed in this study. Table 1 lists the mass percentages that made up the B″s composition. It was prepared using the procedures outlined in the references [32,33]. 

### 2.2. Biological Evaluation of VD3 Solutions and BG+VD3 Thin Films

The cytotoxicity of VD3 solutions was evaluated on NCTC L929 cells (ATCC cell line), cultured in Dulbecco’s Modified Eagle Medium (DMEM) with 10% fetal bovine serum. Cells were seeded at a density of 1 × 10^5^ cells per well in 250 μL culture medium. The cells were incubated at 37 °C (5% CO_2_) for 24 h with the VD3 solutions (concentrations 5, 2, 1, 0.5, 0.25, 0.125, 0.062, 0.031, 0.015 mg/mL). 

The biocompatibility of MAPLE thin films was evaluated on human dermal fibroblasts (HDF) cells, cultured in StemMACS MSC Expansion Media (Miltenyi Biotec, Bologna, Italy). The cells were seeded at a density of 1 × 10^5^ cells per well in 250 μL culture medium. The cells were incubated at 37 °C (5% CO_2_) for 24 h. After removing the supernatant, DMEM (serum-free) medium and a 1 mg/mL MTT solution (Vybrant MTT Cell proliferation, Thermo Scientific, Waltham, MA, USA) were added on top of the cells. The cells were incubated in the presence of 1 mL of MTT solution for 4 h at 37 °C and 5% CO_2_. After 4 h, the formazan crystals formed following the metabolism of the MTT compound were solubilized in an SDS-HCl solution and incubated for 18 h. Afterwards, the optical density proportional to the cellular viability was read at 550 nm (Thermo Scientific SkanIt instrument). The cell supernatant was used to quantify the lactate dehydrogenase (LDH) LDH level (Cytotoxicity Detection kit, Sigma-Aldrich, St. Louis, MO, USA). LDH is an oxidoreductase (E.C. 1.1.1.27) that is present in most organisms. Cells that no longer have membrane integrity release cytoplasm containing this enzyme (LDH) into the culture medium. It is a quantitative test that indicates the number of dead cells. The solution resulting from the reaction can be read spectrophotometrically at 490 nm. For LDH quantification, 100 μL reaction mix was prepared, which contained all the components of the mix equally. Then, 50μL of duplicate medium were collected from the test plate and transferred to a 96-well plate. After adding 100 μL over each sample, the plate was incubated for 15–20 min in the dark. Based on the LDH level in the culture medium, the color intensity of the pink solution varied directly proportional to the number of dead cells in the sample which was read on a spectrophotometer (Flex Station 3) at a wavelength of 490 nm.

Cell morphology was evaluated after 24 h of incubation, using an Olympus IX73 inverted fluorescence microscope (λex/em 488 nm/515 nm and λex/em 570 nm/602 nm). Cells were fluorescently labelled using DAPI (Invitrogen, Carlsbad, CA, USA) and Cell Mask (Invitrogen).

The inflammatory profile of HDFa cells cultured on thin films obtained by MAPLE was analyzed by the ELISA technique after 24 h incubation. The cytokine levels were measured using commercial kits (# EH2IL6 and EHIL10, Invitrogen, Carlsbad, CA, USA) following the manufacturer’s instructions.

### 2.3. Experimental Conditions for MAPLE Transfer

A rotating cryogenic target was evaporated by a UV KrF* pulsed laser beam (25 ns, 248 nm) in a vacuum deposition chamber using the MAPLE processing method, which is a “flexible” laser-based processing technique [34]. The complex organic material of interest was diluted and used to make the target. The material to be deposited was dissolved in a high vapor pressure, light-absorbent solvent. Usually, the UV laser beam damages a variety of substances, including high molecular weight species such as polymeric or organic molecules. By using the cryogenic approach of the MAPLE technique, these types of materials can be softly transferred onto different substrates, without generating heat or causing deterioration.

**Figure 1:**
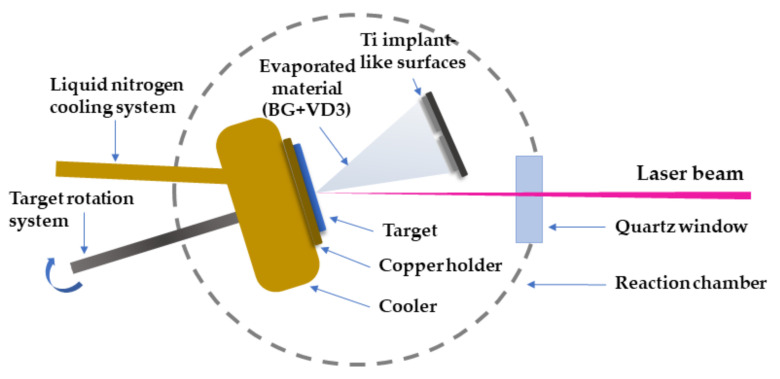
Simplified scheme of the MAPLE experimental set-up.

The principle of the MAPLE experiment is displayed in Figure 1.

To prepare a MAPLE target, the materials of interest (in our case BG57 and VD3) were dissolved in dimethyl sulfoxide (DMSO). The predominant liquid phase of the target (matrix or solvent) was selected so that: (i) it was not toxic in the liquid or vapor phase, (ii) it could form a dilute solution (homogeneous suspension), (iii) the incident laser energy was absorbed only by the solvent molecules and not by those of the material of interest.

The following solutions were prepared for the MAPLE targets:

(a) BG57—Using a magnetic stirrer, 0.08 g of BG powder and 20 mL of DMSO were combined, and the mixture was subsequently homogenized.
(b) Considering the biological assays performed on VD3 before incorporating it into thin films, 0.5, 0.25, 0.125 mg/mL VD3 in DMSO were mixed with 0.08 g of BG57. The obtained mixtures were further denoted as BG57+VD3_05, BG57+VD3_025 and BG57+VD3_0125. A magnetic stirrer was used to homogenize the mixtures.

The solutions thus obtained were poured into a copper plate and then immersed in a liquid nitrogen bath to solidify. The frozen targets were introduced into the vacuum chamber on a cryogenically cooled rotating target holder, which was continuously cooled down to avoid melting during the laser irradiation. At the same time, the targets were kept at the temperature of liquid nitrogen with the help of a cooler, connected to a liquid nitrogen tank, throughout the experiments. During the experiments, the targets were rotated at a frequency of 50 rpm to avoid perforation and to ensure a uniform deposition. 

With a KrF* laser source COMPexPro 205 model (Lambda Physics-Coherent) operating at a repetition rate of 15 Hz, MAPLE depositions were carried out. A laser pulse of 400 mJ energy was focused on a spot with an area of 27 mm^2^. At a 5 cm spacing distance, the target and substrate holder were arranged in a plan parallel configuration. During the deposition of a single coating, the number of laser pulses was 100,000. The ambient pressure inside the stainless-steel vacuum chamber was set at 10^−4^ mbar for all experiments.

### 2.4. Thin Films Characterization Methods

The stoichiometry and chemical functional integrity of thin films were studied by means of Fourier-transform infrared spectroscopy (FTIR). The FTIR study was performed using a Shimadzu IRTracer 100, equipped with an attenuated total reflection (ATR) module, operating in the range 8000–400 cm^−1^ with a resolution of 4 cm^−1^. The measurement was made in absorbance mode and 54 individual scans were recorded for each sample.

The morphological features of the BG57 and BG57+VD3_05, BG57+VD3_025, and BG57+VD3_0125 thin films were investigated by means of an Apreo S ThermoFisher scanning electron microscope (SEM), having a maximum resolution of 0.7 nm. SEM micrographs were acquired in top-view on Ti substrates and in cross-section on Si wafers at a working voltage of 10 kV and pressure of 1 × 10^−3^ Pa. To decrease the electrical charge during the analyses, the samples were coated with a thin gold film.

The surface topography of the BG57 and BG57+VD3_025 samples was obtained using atomic force microscopy (AFM). The scans were performed in tapping mode with a Veeco microscope, on a surface of 10 μm^2^, with a scanning speed of 0.3 Hz and a resolution of 512 pixels; an RTESPA needle was used.

The wettability of the MAPLE coatings was investigated by the sessile drop method, using an Attension Theta Lite (TL) 101 optical tensiometer (version 1.0.3, Biolin Scientific, Vastra Frolunda, Sweden), under atmospheric conditions (22 ± 1 °C, 42% relative humidity). The contact angle between the test liquid and surface of the specimens was measured and the roughness parameters were collected.

### 2.5. Electrochemical Investigation

Electrochemical impedance spectroscopy (EIS) was employed to assess the performance of the investigated samples, by applying a sinusoidal signal of 10 mV amplitude, in a frequency range of 0.1 ÷ 10^3^ Hz. The test was performed using a VersaSTAT 3 Potentiostat/Galvanostat system (Princeton Applied Research, Oak Ridge, TN, USA) coupled to a typical three electrode setup based on a Pt counter electrode, Ag/AgCl (saturated KCl) (0.197 V) reference electrode and the working electrode, which consisted of uncoated Ti, and Ti coated with BG57 (BG57 Ref) or BG57+VD3_025. The testing medium used in the current study was SBF, having a temperature of 37˚C. The samples were immersed for 24 h and VersaStudio software (version 2.60.6, Princeton Applied Research, Oak Ridge, TN, USA) recorded the impedance data after 1, 12 and 24 h of immersion. For the fitting procedure, zView software (version 12136-4, Scribner Associates Inc., Southern Pines, NC, USA) was used.

## 3. Results and Discussion 

The purpose of this study was to obtain thin films based on BG and VD3 to increase the performance of Ti implants at the biointerface. BG are an excellent alternative for enhancing the bioactivity and biocompatibility of implants because they are extremely biocompatible and more likely to integrate with human tissue than the sole metallic implants. The advantages of BG include encouraging tissue regeneration, degrading at a similar rate as tissue regeneration, and replacing broken bone and tissue that will integrate well with the body’s environment [18]. Due to its reaction process in a host biological environment, which results in the development of a layer of hydroxycabonate apatite (HCA) with a mineral composition comparable to bone on the glass surface, BG creates linkages with the host bone, promoting its formation. Moreover, BG has the ability to control or prevent the corrosion of implant metals in biological conditions [35]. Vitamin D is a combination of steroid derivatives. The two most significant forms of vitamin D are vitamin D2-ergocalciferol, which is found in plant-based foods, and vitamin D3-cholecalciferol, which is found in animal-based foods. Additionally, VD3, the biologically active form of vitamin D, is produced in the skin by exposure to sunlight, earning it the name “sunshine vitamin“ [36]. Moreover, by stimulating osteoblasts and osteoclasts, VD3 also controls bone mineralization and metabolism [37,38]. Depending on the dosage, VD3 can either speed up or slow down the production of new bones [38]. By encouraging intestinal absorption of calcium and phosphate, vitamin D_3_ (VD3), is crucial for maintaining mineral homeostasis [39].

### 3.1. Biological Evaluation of the VD3 Solution Cytotoxicity before Incorporation into Thin Films

**Figure 2:**
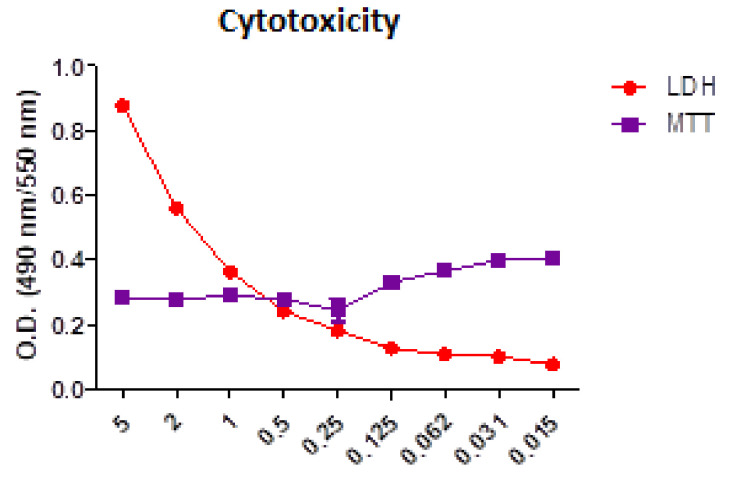
The cytotoxicity of VD3 solutions on NCTC L929 cells—comparative analysis of MTT and LDH tests; *X*-axis—VD3 concentrations (mg/mL).

The comparative analysis of the MTT and LDH test results for the VD3 solutions are shown in Figure 2. A range of VD3 concentrations were tested to select the optimal formulation to be used in subsequent experiments.

It can be observed that VD3 solutions become cytotoxic at concentrations higher than 0.5 mg/mL.

### 3.2. Surface Investigation of As-Deposited Thin Films

The stoichiometry and chemical functional integrity of thin films before and after MAPLE deposition were analyzed by FTIR in order to observe if any changes occurred in the composition of the thin films. 

**Figure 3:**
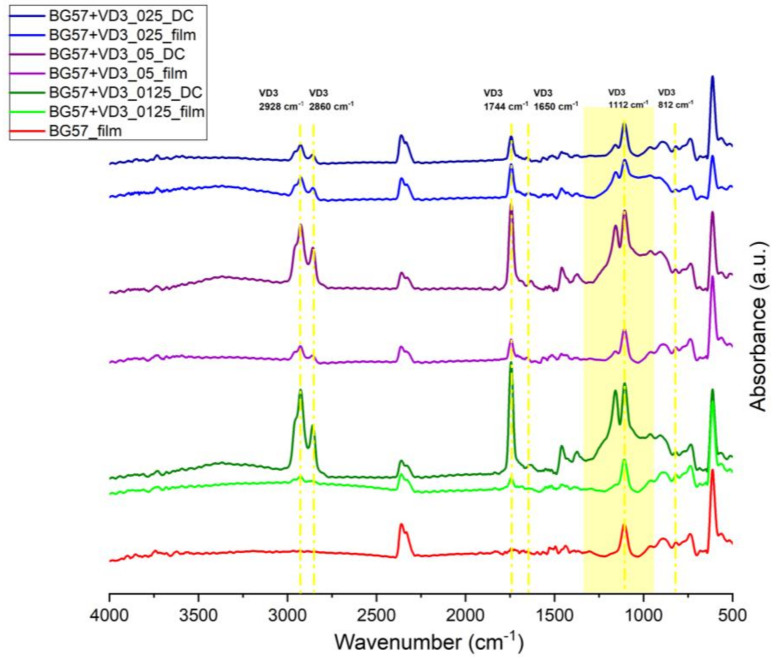
FTIR spectra obtained for BG+VD3 thin films (_film) and drop-casts (_DC).

The spectral characterization of materials of interest dissolved in DMSO does not indicate structural changes during the freezing process nor after the target’s irradiation; this validates the use of the MAPLE technique for obtaining quality composite coatings. A confirmation of the bioactive thin films’ deposition is related to the presence of bands specific to the functional groups of BG57 and VD3 in the FTIR spectra (Figure 3).

In the FTIR spectrum of BG57+VD3 films (Figure 3), the peak corresponding to the value of 1650 cm^−1^ indicates the presence of double bonds between C atoms, of which VD3, having the name cholecalciferol, has four. The band located at 812 cm^−1^ corresponds to the presence of aromatic rings, especially an alkane ring with six C atoms with three substitutions, of which the VD3 molecule has two in its structure. Between 1310 and 1000 cm^−1^, the bands corresponding to C-C and C-O bonds can be found, which are related to aliphatic chains and a hydroxyl group that form the molecular structure of VD3 [40]. At the same time, cholecalciferol is characterized by the asymmetric CH_3_ and symmetric CH_2_ stretching modes at 2928 cm^−1^ and 2860 cm^−1^, respectively [41]. Two other characteristic peaks are found around the values of 1744 and 1112 cm^−1^, representing the stretching vibrations of C=O and C-O-C bonds, respectively. Since BG was deposited as a thin film together with VD3, the presence of Si–O–Si groups can also be observed [40,42]. However, from the point of view of the efficient transfer of the composite material and the preservation of chemical integrity, optimal results were revealed for the BG+VD3_025 samples.

SEM provides valuable information for implant biocompatibility and potential applications for bone repair, by allowing researchers and clinicians to visualize the surface morphology and structure of biomaterials, implants, and tissues at the micro and nano-scale. For bone repair, SEM can reveal the texture and roughness of the coating on implants’ surfaces, which can influence osseointegration. Micro- and nano-scale surface features are particularly important because they can affect cell adhesion and tissue response. 

**Figure 4:**
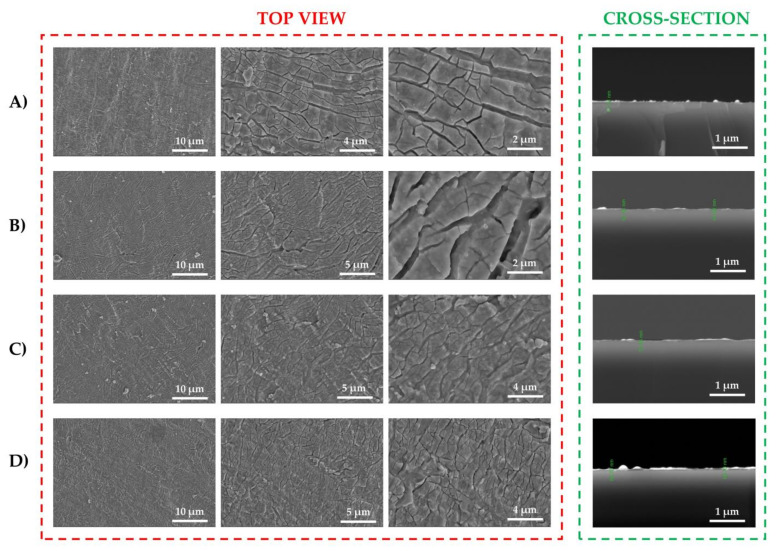
SEM images of MAPLE thin films deposited on Ti substrates in top view (red square) and cross-section modes (green square), at different magnifications: (**A**) BG57, (**B**) BG57+VD3_05, (**C**) BG57+VD3_025 and (**D**) BG57+VD3_0125.

The micro-topography of the BG57+VD3 thin films is observable from SEM images, at different magnifications, as depicted in Figure 4 (red square). A morphology consisting of a dense matrix with many irregularities is noted, and more pronounced for the BG57 sample. This type of surface favors a good adhesion of bone cells, and consequently, for bone repair applications. [43]. Irregularities on the surface, such as those observed in the dense matrix, increase the overall surface area. This provides more opportunities for cells, to adhere to the material [44]. An extended surface area can lead to improved cell attachment, proliferation, and migration, which are essential for tissue regeneration [45]. Irregularities on the surface can enhance the osteoconductive properties of the material; can serve as sites for initial cell attachment and can facilitate the deposition of bone minerals, leading to the formation of new bone around an implant [46]. It seems that the morphology of the film surface changes with the addition of VD3; the surface becomes smoother. Taking into account the SEM microscope’s magnification and software, the approximate thickness of the thin films was calculated to be around 57 µm (with an error margin of ±5%), as depicted in Figure 4 (green square).

### 3.3. Biological Evaluation of the MAPLE-Deposited Thin Films

**Figure 5:**
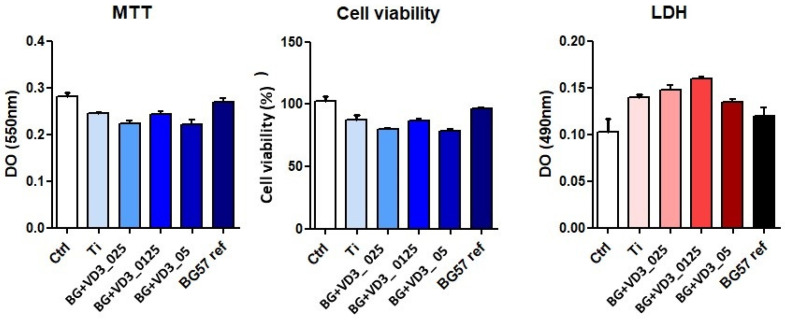
(**left**) The biocompatibility of MAPLE thin films tested on HDFa cells: (**center**) the results of the MTT test; Conversion of DO values to percentages of cell viability of MAPLE films; (**right**) The results of the LDH test (**right**).

Following the incubation with human cells, BG57 and BG57 +VD3_ 025 thin films showed the highest degree of biocompatibility, as illustrated by the MTT assay values (Figure 5a), which were similar to the control sample (unstimulated cells, under standard culture conditions—MTT DO = 0.287 ± 0.02). No significant values were found for the BG+VD3_025 (DO_550nm_ = 0.266 ± 0.01), BG+VD3_05 (DO_550nm_ = 0.234 ± 0.1), BG+VD3_0125 (DO_550nm_ = 0.249 ± 0.12) composites compared to the Ti control (DO_550nm_ = 0.277 ± 0.12) and the BG57 reference (DO_550nm_ = 0.282 ± 0.11). 

The conversion of OD values into cell viability percentages (Figure 5b) showed that all tested materials are biocompatible, with cells having a viability level greater than 80%. 

According to the LDH investigation (Figure 5c), all tested materials had higher values compared to the control sample (unstimulated cells). For control cells, the LDH values were lower compared to the tested composites (DO_490nm_ = 0.105 ± 0.12). Even though all the composites lead to higher LDH release (BG+VD3_025-DO_490nm_ = 0.137 ± 0.02), BG+VD3_05 (DO_490nm_ = 0.126 ± 0.1), BG+VD3_0125 (DO_490nm_ = 0.139 ± 0.11) this was not statistically significant compared to the Ti control (DO_490nm_ = 0.105 ± 0.12) and the BG57 reference (DO_490nm_ = 0.122 ± 0.16). 

From all tested thin films, the BG57 sample presented the highest degree of biocompatibility, having increased values for the MTT test (which measures cell viability) and the lowest values for LDH (a test that quantifies cell death). 

**Figure 6:**
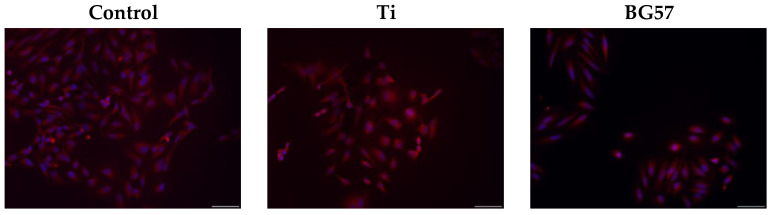

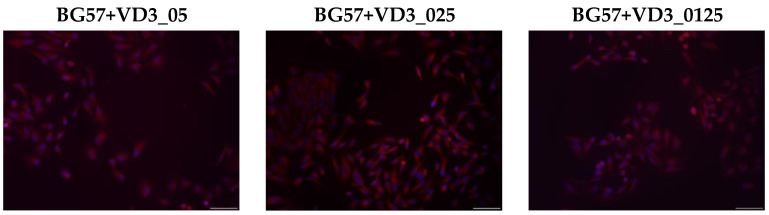
Morphology of HDF cells grown on uncoated and MAPLE-coated Ti substrates (magnification bar 200 µm).

The evaluation of cell morphology (Figure 6) was performed by fluorescence microscopy following cultivation of human cells on the obtained thin films for 24 h. After incubation, cells were fixed with methanol for 20 min and stained with DAPI (1 μg/mL; blue) and Cell Mask (red dye for cell membrane).

The cultivation of human cells on the thin films did not induce major cellular changes. Cells cultured on the BG57+VD3_025 sample had similar morphology to that of unstimulated control cells. 

**Figure 7:**
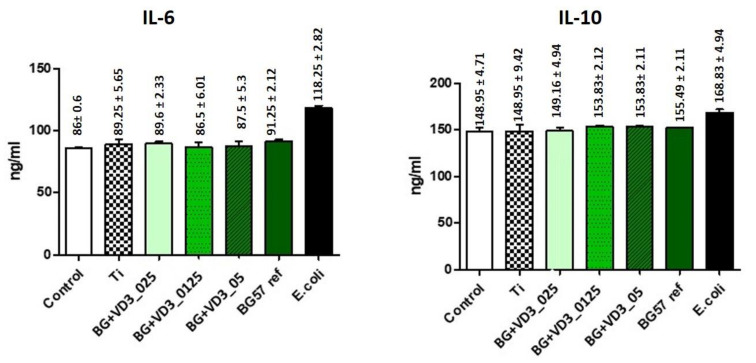
IL-6 and IL-10 levels quantified by ELISA on HDF cells; stimulation with *E. coli* represented the positive control.

The inflammatory profile of human cells cultured on thin films obtained by MAPLE was analyzed by the ELISA technique. After 24 h of incubation, the level of interleukin (IL)-6 (Figure 7 left) and IL-10 (Figure 7 right) in the cell supernatant was quantified. 

It was observed that the thin films did not change the pro- and anti-inflammatory profile of the HDF cells, the IL-6 and IL-10 levels being similar to those of the control sample (represented by cells cultivated in standard, unstimulated conditions).

Considering the positive biological results of the BG+VD3_025 thin films, corroborated with FTIR analyses, this particular thin film was considered for further investigations.

### 3.4. AFM and Contact Angle Measurement

AFM plays a critical role in characterizing the surface properties of biomaterials intended for implant biocompatibility applications. It helps researchers understand how materials interact with bone cells at the nanoscale, allowing for the design and optimization of biomaterials that promote cell adhesion, proliferation, and differentiation, ultimately facilitating bone tissue regeneration and repair. 

In Table 2 both 2D and 3D images of the BG and BG+VD3_2 surfaces, as well as roughness parameters obtained for each individual surface can be seen. The surface of the films is not smooth, the root mean square roughness (R_rms_) falling within the range of 80–130 nm. The surface roughness can be correlated with the SEM morphology, noting that the coatings containing a more textured surface led to obtaining surfaces with a higher roughness. A rough or textured surface can promote better cell adhesion, proliferation, and differentiation, which are critical for bone tissue regeneration applications [47]. Cells, such as osteoblasts in bone repair applications, tend to adhere better to surfaces with micro- and nano-scale features [48]. These features mimic the natural extracellular matrix (ECM) and provide anchoring points for cells to attach and proliferate [48]. Implants with moderately rough surfaces encourage tissue integration. When the implant’s surface texture resembles natural tissue structures, it enhances the integration of the implant with surrounding tissues [49]. This is especially critical for orthopedic implants such as hip or knee prostheses. Surface roughness can facilitate the adsorption of proteins from the surrounding biological environment [50]. Proteins are essential for mediating cell-surface interactions. A rougher surface area can accommodate more proteins, improving cell signaling and attachment [51]. Although the surface of the samples showed irregularities, the thin films completely covered the substrates. The presence of voids was not observed. 

**Figure 8:**
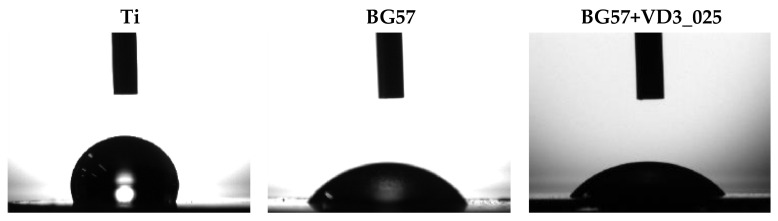

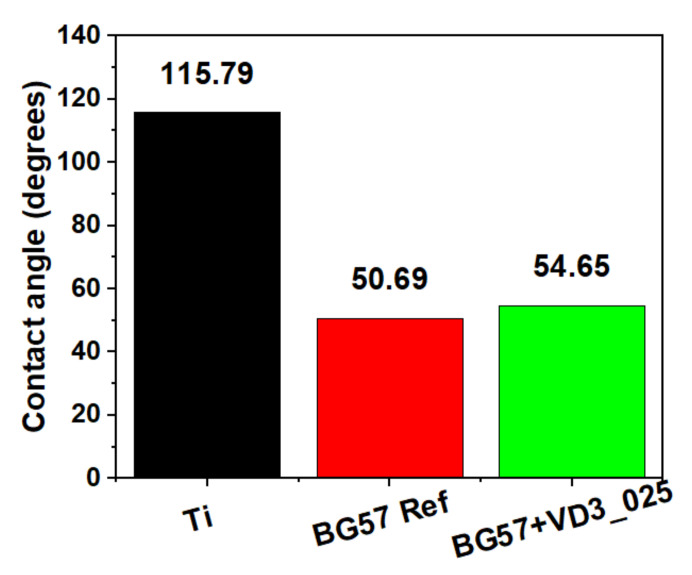
Surface wetting properties of investigated samples.

The first step in the development of new tissue is cell attachment, which is closely related to how wettable a biomaterial’s surface is [52]. In the case of hydrophilic surfaces, cell affinity and the rapidity of cell spreading could be enhanced [53]. The wetting properties of the surfaces were highlighted by measuring the contact angle between the solid surface (BG57 and BG57+VD3_025) of the investigated material and a liquid medium (SBF) (Figure 8).

The sample coated with BG57 achieved a significant improvement in hydrophilicity with a contact angle of 50.69°. Instead, as can be seen in Figure 8, after applying the BG57+VD3_025 thin film, the contact angle increased to a value of 54.65°. The addition of VD3 significantly improved the hydrophilicity of the Ti surface. 

The contact angle of a surface is influenced by various surface properties, with surface roughness being a commonly studied factor [54]. However, it is important to note that factors other than roughness contribute to the ultimate contact angle value. These factors encompass surface hardness, plasticity threshold, microstructure, crystallinity, and grain size [55]. The mode of interaction of the solid surface with a liquid medium is closely related to the terminal groups of the molecules at the surface interface, which can be hydrophilic or hydrophobic [56]. VD3 possesses one polar hydroxyl group, even though it is thought to be a fat-soluble vitamin due to the dominance of the non-polar hydrocarbon region. The hydroxyl group found in the chemical composition of VD3 may be responsible for the decrease in the water contact angle for the thin films containing VD3. The broad band at about ~3200 cm^−1^ in the FTIR spectrum for the sample showed the presence of an OH functional group [57].

### 3.5. Electrochemical Performance of the Tested Samples

EIS was employed for monitoring the corrosion process over 24 h of immersion in SBF, since its “non-destructive” character can give a valuable insight into the electrochemical reactions occurring on the surface, especially when in-time predictions based on successive measurements are targeted. 

**Figure 9:**
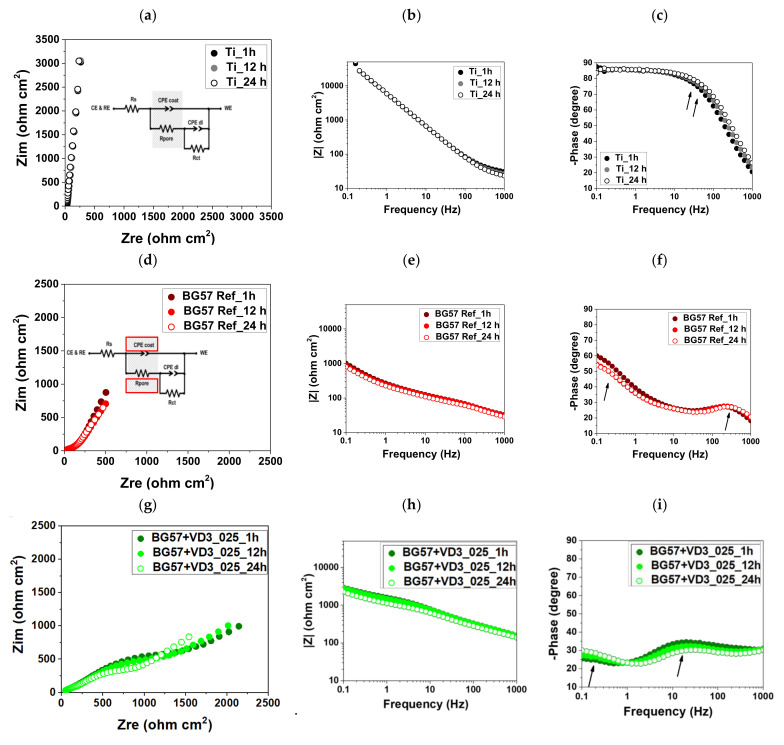
Nyquist (**a**,**d**,**g**) and Bode amplitude (**b**,**e**,**h**) and phase plots (**c**,**f**,**i**) for Ti, BG57 Ref and BG57+VD3_025% obtained after 1, 12 and 24 h of immersion in SBF at 37 °C (Rs = the solution resistance, CPEcoat = coating capacitance, Rcoat = resistance associated to the current flow, CPEdl = double layer capacitance, Rct = charge transfer resistance).

The impedance data obtained for the investigated samples after different immersion times (1, 12 and 24 h) in a SBF environment, are presented as Nyquist (Figure 9a,d,g) and Bode amplitude (Figure 9b,e,h)/phase graph (Figure 9c,f,i). Although the presence of a semicircle can be observed in the Nyquist plot, which is indicative of a single time constant, there is evidence of the formation of two-phase angles (indicated by arrows) in the medium and high frequency ranges in the Bode phase plot (Figure 9c,f,d). This is more visible in the case of the investigated coatings. Milošev et al. proved by XPS that there is a passive film formation on top of Ti alloys immersed in physiological solution, mainly composed of Ti oxides and suboxides [58]. Analyzing the impedance plots obtained in the current study, one can note a convolution of the two phases, without a visible differentiation in the frequency range measured. Similar results were obtained by Gugelmin et al. [59], where for the fitting of the impedance data, a two-time electrical equivalent circuit (EEC) was used in order to take into consideration either the investigated coatings or the layer formed on the surface of Ti (in accordance with the bilayer oxide model [60]). Based on these results, characteristic electrical components were used to model the impedance data to obtain quantitative information, the used EEC being presented as an inset in Figure 9. At a microscopic level, a defect free surface condition characteristic of a pure capacitor is not accomplished, multiple structural features being present in real life (surface disorder, inhomogeneity, geometric irregularities, roughness, or porosity of the working electrode) [61]. Therefore, since the current flow can have a non-uniform distribution generated by the mentioned defects, a CPE-like behavior (constant phase element) can be a more realistic electrical element used to simulate the coating and the double layer capacitance in the used EEC (CPEcoat and CPEdl, respectively) [62,63]. Rs describes the solution resistance, Rcoat is the resistance associated with the current flow, whereas Rct represents the charge transfer resistance. Following the above presented concluding remarks, a deductive approach was further used, by fitting the experimental impedance data to have an insight into the physical reactions occurring at the material–electrolyte interfaces.

The best-fit values of the electrochemical parameters obtained for the impedance curves recorded after 1, 12 and 24 h of immersion in SBF at 37˚C are presented in Table 3, along with χ2 parameter, which was used to validate the fitting procedure. Since, in this case, the obtained values proved to be around 104–103, which means that there is a good agreement between experimental and simulated impedance curves, the assumed physical behavior can be considered as rational and the electrochemical interpretation can be further carried out. A comparative assessment of the systems under investigation allowed not only to observe the electrochemical tendency as a function of immersion time, but also to evaluate the protective performance of the coatings according to their composition. Based on the presented results, one can note only a slight decrease recorded over an immersion period of 24 h in a SBF environment, behavior which was exhibited by all samples. Even though Ti alloy showed a high phase angle, indicating almost ideal dielectric properties, the low Rpore value displayed by the formed oxide layer can be accounted for providing valuable information related to its porous structure. Furthermore, a different behavior was promoted when BG type coatings were used which was more evident when VD3_025% was added to the BG57 Ref, which showed a high improvement in the Rpore parameter. 

## 4. Conclusions

The study successfully demonstrated the feasibility of using MAPLE to deposit composite coatings of BG and VD3 on Ti implant-like surfaces. The coatings exhibited promising properties, including enhanced biocompatibility, improved hydrophilicity, and good corrosion resistance.

Our coatings hold promising perspectives for several targeted applications in the field of biomaterials, tissue engineering, and regenerative medicine. When used as coatings on orthopedic implants, they can promote osseointegration, which is the direct bonding of bone with the implant. This property is especially valuable in joint replacements, dental implants, and spinal fusion surgeries. MAPLE synthesized thin films offer the potential for controlled drug delivery systems. VD3 is important for bone health and can be released slowly from the coating to promote bone growth around the implant, i.e., osseointegration. This is particularly beneficial in cases where patients have deficiencies in vitamin D or require additional supplementation for bone healing. BG coatings, with or without VD3, can be engineered to have antimicrobial properties. This can help reduce the risk of implant-related infections, which are a significant concern in various medical devices, including joint prostheses and dental implants. BG+VD3 thin films can be tailored for use in orthopedic implants, such as hip and knee prostheses. These thin films can enhance the longevity of the implant and reduce complications such as implant loosening or infection. In the case of osteoporosis, where bone density is reduced, coatings that release VD3 could potentially aid in improving bone health and reducing the risk of fractures. Thin films that combine BG with VD3 may offer synergistic effects, providing both structural support and biological cues for bone regeneration.

The perspectives for these applications depend on ongoing research and development efforts to optimize coating composition, thickness, and drug release kinetics. Additionally, clinical trials and long-term studies are necessary to validate the safety and efficacy of these coatings in various medical contexts. Nevertheless, the BG+VD3 thin films hold significant potential for improving patient outcomes in a range of musculoskeletal and regenerative medicine applications. 

However, one significant challenge of our laser deposition technique is its limited scalability for coating large items such as hip prostheses or other substantial medical implants. While MAPLE offers advantages in terms of precision and the ability to deposit thin films with unique properties, it is typically better suited for coating smaller surfaces. 

## Figures and Tables

**Table 1:** Composition of BG57 powder used in the MAPLE experiments.

Oxides (wt%)						
BG	SiO_2_	Na_2_O	K_2_O	CaO	MgO	P_2_O_5_
	56.5	11	3	15	8.5	6

VD3 was purchased from Sigma-Aldrich Chemie GmbH (Steinheim, Germany).

**Table 2:** Two-dimensional and three-dimensional AFM images of the sample surface recorded on 10 µm^2^ surfaces and the roughness parameters.

Sample	2D Image	3D Image	Roughness Parameters (nm)	
			Ra	R_rms_
BG57	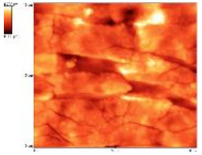	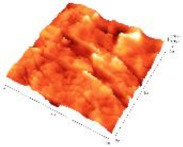	80.3	103.4
BG57+VD3 _025	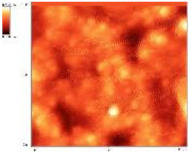	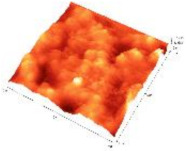	62.1	79.4

**Table 3:** EIS fitted parameters obtained after 1, 12 and 24 h immersion in SBF at 37˚C.

Electrochemical Parameters	Time	Samples		
		Ti	BG57 Ref	BG57+VD3_025
Rs (Ω cm^2^)	1 h	27	22	56
	12 h	23	19	52
	24 h	19	15	42
Qcoat (μF s^(α−1)^ cm^−2^)	1 h	19.89	294.34	147.95
	12 h	19.95	301.59	175.39
	24 h	20.25	486.12	226.97
αcoat	1 h	0.96	0.61	0.48
	12 h	0.96	0.60	0.45
	24 h	0.96	0.54	0.43
*Rpore* (Ω cm^2^)	1 h	87	132	3047
	12 h	75	123	3057
	24 h	70	130	2163
*Q*dl (μF s^(α−1)^ cm^−2^)	1 h	9.90	1193.00	806.34
	12 h	9.68	1501.90	634.33
	24 h	9.13	1427.60	577.22
αdl	1 h	0.93	0.68	0.90
	12 h	0.93	0.63	0.90
	24 h	0.93	0.66	0.90
*R*ct (Ω cm^2^)	1 h	-	-	5076
	12 h	-	-	6014
	24 h	-	-	4304
*χ^2^*	1 h	2 × 10^−4^	8 × 10^−4^	8 × 10^−4^
	12 h	2 × 10^−4^	7 × 10^−4^	1 × 10^−3^
	24 h	2 × 10^−4^	8 × 10^−4^	9 × 10^−4^
